# Perception of Odors Linked to Precise Timing in the Olfactory System

**DOI:** 10.1371/journal.pbio.1002021

**Published:** 2014-12-16

**Authors:** Michelle R. Rebello, Thomas S. McTavish, David C. Willhite, Shaina M. Short, Gordon M. Shepherd, Justus V. Verhagen

**Affiliations:** 1 The John B. Pierce Laboratory, New Haven, Connecticut, United States of America; 2 Yale School of Medicine, Dept. Neurobiology, New Haven, Connecticut, United States of America; The Rockefeller University, United States of America

## Abstract

While the timing of neuronal activity in the olfactory bulb (OB) relative to sniffing has been the object of many studies, the behavioral relevance of timing information generated by patterned activation within the bulbar response has not been explored. Here we show, using sniff-triggered, dynamic, 2-D, optogenetic stimulation of mitral/tufted cells, that virtual odors that differ by as little as 13 ms are distinguishable by mice. Further, mice are capable of discriminating a virtual odor movie based on an optically imaged OB odor response versus the same virtual odor devoid of temporal dynamics—independently of the sniff-phase. Together with studies showing the behavioral relevance of graded glomerular responses and the response timing relative to odor sampling, these results imply that the mammalian olfactory system is capable of very high transient information transmission rates.

## Abstract 2

The temporal dynamics of glomeruli activity can be behaviorally discerned by mice down to 13 milliseconds.

## Author Summary

Olfactory receptor neurons respond to odors in the olfactory epithelium located in the nasal cavity in mammals. Each olfactory receptor neuron expresses only one olfactory receptor, out of several hundred encoded in the mammalian genome. Olfactory receptor neurons expressing the same olfactory receptor are scattered throughout the olfactory epithelium; however, their axons converge in one of thousands of glomeruli in the olfactory bulb. The glomeruli are the first neural relay station in the olfactory system, where olfactory receptor neurons transmit olfactory information to mitral cells. It is well established that different odors evoke different spatial patterns across the glomeruli. It is believed that the more similar the patterns, the more similar the evoked odor perceptions. Glomeruli also are activated in odor-specific sequences in time. These dynamics could increase the amount of information about odors by immense amounts. We used transgenic mice, whose mitral cells were made responsive to light, and asked how well they could discriminate the temporal dynamics of simple spatial patterns of light presented to the olfactory bulb after each sniff. Mice could detect the presence of temporal dynamics down to 13 ms, which provides ample resolution for them to be able to detect the dynamics in response to actual odors. Mice could also discern whether virtual odors, based on actual olfactory bulb activity, were dynamic or static and did so without reference to exact sniff-time. We conclude that both the spatial glomerular activity patterns and the temporal dynamics thereof are used in the mammalian olfactory system to encode odors.

## Introduction

Different odor stimuli are represented by different spatial patterns of activated olfactory glomeruli in the olfactory bulb (OB), as first shown by activity markers [1]–[3] and supported by the projection patterns of olfactory receptor cells [3]–[5]. Subsequent studies have suggested that these odor patterns are dynamic, evolving over time [6]–[9]. Temporal patterns of glomerular activation reliably differ across glomeruli and depend on the orthonasal odorant and its concentration in anesthetized mice [8],[10],[11] and have also been reported in awake mice [12],[13]. The unfolding of this dynamic odor map occurs by sequential activation of glomeruli at timescales of 10–200 milliseconds [8], and these temporal patterns of activation in the periphery can be read by downstream central brain areas, such as the piriform cortex [14].

The behavioral relevance of precise olfactory timing has been demonstrated [13],[15], relative to sniffing, in accord with the finding that mitral/tufted cell (MTC) activity relative to sniffs carry significant odor information [16]. These behavioral studies stimulated the olfactory epithelium (OE) with a single optical fiber. However, it remains unknown if sniffing is a necessary timing reference for precise temporal olfactory discriminations or if such discriminations can be performed independently of the sniff cycle using strictly across-glomerular "internal" timing. To assess this possibility, it is necessary to precisely control the spatial and temporal activity across the spatially convergent OE neural terminals at the olfactory bulb glomerular input or their MTC projections. We interrogated optogenetic mice with a novel, custom-designed light projector to afford this multidimensional control.

We used three paradigms to address the hypothesis that mice utilize spatial and temporal patterns of MTC activity to distinguish odors. We found that mice could discriminate between eight light spots that were projected either simultaneously or with internal delay (referenced to glomerular activity irrespective of exact sniff timing) onto the olfactory bulb. A single presentation per trial (Paradigm 1) yielded a delay detection threshold of 150 ms. Multiple sniff-triggered presentations (Paradigm 2) yielded a threshold of 13 ms. In Paradigm 3, mice successfully discriminated a dynamic virtual odor based on an optically imaged OB odor response from the same virtual odor devoid of dynamics, irrespective of the onset times' relation to the sniff-phase. Odors are hence not only encoded but can also be perceptually decoded in a spatiotemporal manner, both with and without reference to sniffing.

## Results

### Paradigm 1: A Single Optical Stimulus per Trial Yields Poor Temporal Discrimination

**Figure 1:**
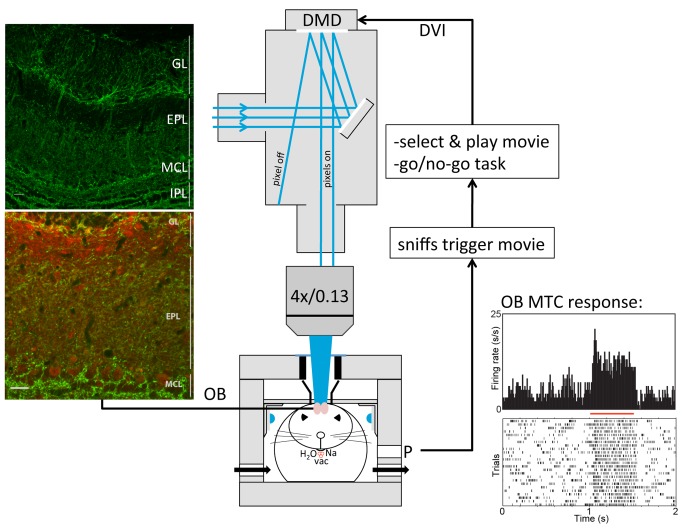
Experimental set-up for odor-movie discrimination. **Left**, ChannelRhodopsin (ChR2) immunocytochemistry of olfactory bulb (OB, top), showing Yellow Fluorescent Protein (YFP) expression in the glomerular layer (GL) and mitral cell layer (MCL). Colocalization of mitral cell marker, 5HT2A (red) and YFP (bottom). Scale bar 20 µm. **Middle**, schematic of the experimental setup. A Digital Micromirror Device (DMD), steered via a Digital Video Interface (DVI), projects movies onto the dorsal OB of the head-restrained thy-1 ChRhod1 mouse located in a whole-body pletysmograph via a sealed "light-funnel." Masking LEDs were located next to each eye. A three-way lick spout presented water as reward or 1M NaCl as punishment. A vacuum cleaned the spout between trials. **Right**, two PCs were used to run the setup. One PC detected sniffs which triggered another PC to generate and display the movies on the DLP. **Bottom Right:** Extracellular single unit in vivo electrophysiological recording from the mitral cell layer. MTC evoked activity was time-locked to the light stimulus (red bar) presented at brightness levels comparable to that used in the behavioral study (top panel shows a Post Stimulus Time Histogram (PSTH) and bottom panel shows a raster plot of firing patterns).

**Figure 2:**
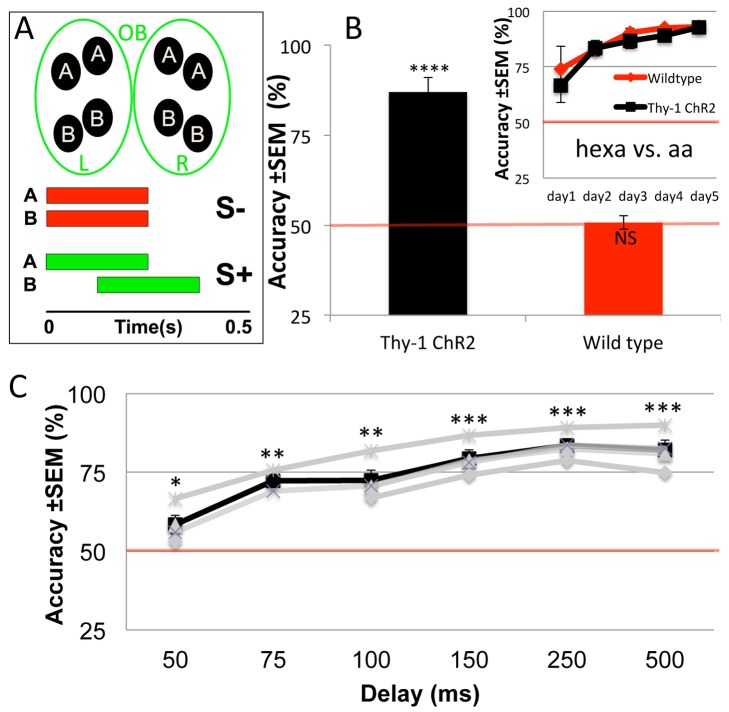
Behavioral Paradigm 1: a single model movie per trial. (**A**) Schematic of S+ and S- patterns projected onto the OB. Anterior ("A") and posterior ("B") spots roughly match the size of large Regions Of Interest (ROIs) typically imaged from the bulb and mimic internal symmetry of each bulb. Only the S+ movie is dynamic, in which the posterior "B" spots are presented after "A". Time-integrated brightness is the same for all conditions. (**B**) Performance of Thy1-ChR2 and wild-type mice on light discrimination task. Inset, Thy1-ChR2 and wild-type mice show accuracy over 80% on an odor discrimination task within 3 d from the start of training (hexanone [hexa] versus amyl acetate [aa]). (**C**) Trace showing average performance of Thy1-ChR2 mice (*n* = 4) with decreasing latencies between the “A” and “B” patterns. Mice are able to perform above 75% accuracy at a threshold of 150 ms. Each grey trace represents a mouse; the black trace is the average. Note non-linear *x*-axis. (*** *p*<0.0001, ** *p*<0.0005, * *p*<0.05, one-sided unpaired *t*-test for above 50%.)

The experimental animals used were Thy-1 ChR2 mice, which express ChR2 in the MTCs of the OB (Fig. 1) [17],[18]. We opted for post-synaptic targets and, hence, for bypassing the inputs to the OB (and the sensory activation of processing in the glomerular layer), to definitively show that when we controlled the timing of MTCs directly, this timing information would be relayed to downstream targets, effectuating the behavioral decisions. Head-fixed Thy-1 ChR2 and control C57BL6 mice were first trained on a go/no-go task to distinguish between two odors (0.1% amyl acetate and 0.5% 2-hexanone) (Fig. 1). Mice were water restricted and rewarded with a drop of water for correctly licking for the S+ stimulus. An incorrect lick during a S- trial was punished with a drop of 1M NaCl. Mice usually took 1–3 d to acquire the odor discrimination task and performed at >80% accuracy (87.0%±1.8% *n* = 4) (Fig. 2B, inset). They were then switched to a go/no-go task with OB light patterns as the stimulus; i.e., they were required to discriminate between two light patterns that were spatially identical but differed temporally (i.e. temporal discrimination). The S- stimulus consisted of two sets of four ellipsoid bright spots, each spot mimicking clusters of glomeruli (see Methods for dimensions and S1 Text for further biomimetic design constraints). Four ellipses were presented at the rostral part of the dorsal OB (“A,” Fig. 2A), the other set at the caudal part (“B,” Fig. 2A). The S+ stimulus consisted of the identical spatial pattern, however the onset of the two caudal sets of four ellipses was delayed by a specific interval that could be varied manually or automatically (Fig. 2A). The duration of each spot was 250 ms. The overall intensity over time was therefore identical between S+ and S- stimuli, ensuring that whatever change was being detected by mice was purely due to timing differences.

Access to the OB was provided by creating an optical window by thinning the bone over the dorsal OB. Light patterns (3×4 mm) were projected over the OB using a Digital Light Processor (DLP) projector (Texas Instruments). The DLP enabled us to project high-resolution spatiotemporal movies at 1024×768 pixels (3.9×3.9 µm per pixel, thus a glomerulus spanning a diameter of approximately 25 pixels), 1,440 (binary) frames per second onto the mouse OB (Fig. 1).

In Paradigm 1, the mice had to discriminate between the dynamic S+ and static S- stimuli, wherein the S+ stimulus differed in that the onset of the posterior two sets of ellipses was delayed initially by 500 ms (Fig. 2A). A single stimulus was presented at the start of a trial, without respect to sniff times (i.e., not triggered by sniffing). Control wild-type mice were unable to perform this go/no-go task above chance performance (50.8±1.1% *n* = 3, mean ± SEM, *p* = 0.26, above 50%, one tailed unpaired *t*-test, *n* = 3, 10th session), while Thy1-ChR2 mice performed with an accuracy of over 85% (87.0%±1.8% *n* = 4, *p*<0.00001, one tailed unpaired *t*-test, *n* = 4, 11th session) (Fig. 2B). Thy1Chr2 mice were able to do the discrimination with over 75% accuracy within 7 d on average. Once the mice were able to discriminate at an accuracy of 80% for 20 trials (1 block) or 75% accuracy for 2 blocks (*p*<0.005, binomial statistics), the delay was manually decreased from 500 ms by 100–25 ms delays. We found that Thy1-ChR2 mice could successfully discriminate between the stimuli down to a threshold of 150 ms (79.4±3.9%, *p*<0.000001, above 50%, one tailed unpaired *t*-test, *n* = 4) with an accuracy of over 75% (Fig. 2C). Beyond 150 ms, their performance fell below 75% but was still significantly above chance at 50 ms (*p*<0.01). These results showed that Thy1-ChR2 mice could detect temporal differences in the singular (one stimulus per trial) activation of MTCs in the OB, with a temporal resolution of 150 ms.

### Paradigm 2: Sniff-Triggered Multiple Optical Stimuli per Trial Yield 13 ms Resolution

**Figure 3:**
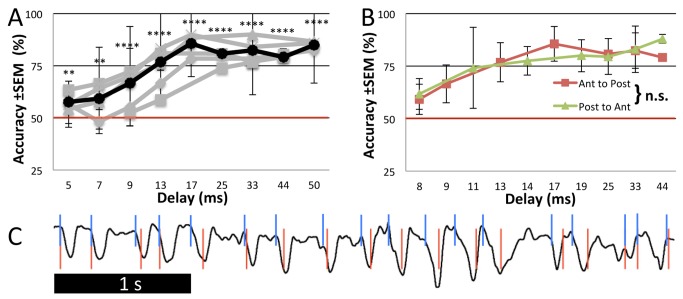
Behavioral Paradigm 2: sniff-triggered model movies. (**A**) Performance of mice with light patterns projected in anterior to posterior order. Each gray trace represents a mouse; the black trace is the average. At 13 ms, on average, mice performed above 75% accuracy (*p* = 0.000004) and this was considered their threshold. (**B**) Comparison of the average performance of mice with the order of light patterns projected either anterior to posterior (*n* = 4) or posterior to anterior (*n* = 3). Note non-linear *x*-axes. n.s.: not significant. (**C**) Sniffing (black line) and sniff inhalation start times (blue lines). Movies were started 10 ms after sniff start times in this paradigm. In Paradigm 3 an intentional jitter was used between sniff inhalation onset and movie onset times (red lines).

The coupling of olfactory responses to respiration is a well-known phenomenon [19] and implies a possible role of the sniff cycle in odor coding. Work by Rinberg and colleagues [13],[15] showed that the timing of odor activation relative to the sniff cycle is an important cue used behaviorally by mice. Therefore, we next introduced the sniff-triggering of light patterns in Paradigm 2. Sniffing was measured using a non-invasive whole body plethysmograph [20], and the same model stimulus movies from the Paradigm 1 were presented approximately 10 ms after each sniff, now for a shorter, 100 ms duration (plus the automatically adjusted "A"–"B" delay, Fig. 2A). Fig. 3C shows an example sniff trace and sniff inhalation onsets.

As expected, mice were able to perform the task much more accurately with a temporal resolution down to13 ms (*p*<0.0001, *n* = 5 mice, seven to ten sessions per mouse) (Fig. 3A). On average, these mice performed above chance at 7 ms (*p*<0.05) and, more strictly, at 9 ms (*p*<0.001), and the average went above 75% (76.8±11.2%) at 13 ms. This suggests that the olfactory system is able to use timing information contained in the MTC response and can do so with a resolution of 13 milliseconds.

The discriminations under Paradigms 1 and 2 could be based on the difference between stimulus element onset times (A versus B latency) and/or overall stimulus duration (A duration + latency), but not overall activity (each stimulus has an identical Area Under the Curve [AUC]). Irrespective of the mechanism, our results point to a high temporal resolution of discriminability of activity among MTCs.

Wachowiak et al. [8],[21] showed that odor response latencies in the OB are regionally organized, with glomeruli in the caudo-lateral OB showing shorter latencies than those antero-medial. Reversing the order of the light patterns, so that the caudal ellipsoids preceded the rostral ones, did not produce any significant difference in mice behavior, showing that the order of activation did not affect their temporal resolution (Fig. 3B) (*n* = 3 mice, 3–12 sessions per mouse).

### Paradigm 3: Mice Can Discriminate Temporal Information in Neural OB Response Patterns

**Figure 4:**
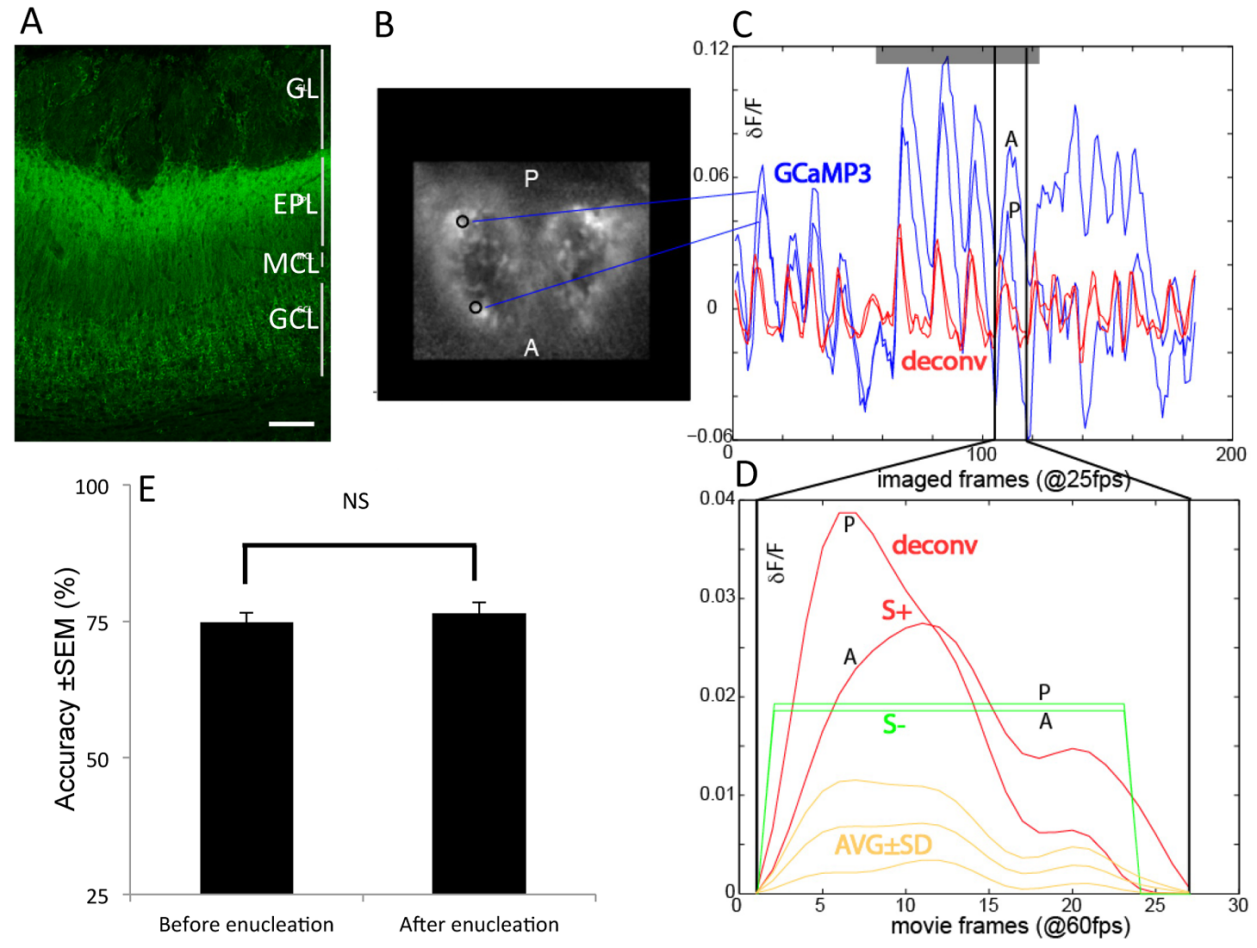
Behavioral Paradigm 3: sniff-triggered playback of a calcium response optically imaged from the dorsal OB of GCAMP3-EMX mice. (**A**) Immunofluorescence of the OB of the GCaMP3-EMX mouse. GCaMP3 expression is seen in sub-populations of periglomerular cells and in the superficial granular layer. Scale bar 100 µm. (**B**) Map of the time-integrated calcium signal outlining an anterior ("A") and posterior ("P") ROI whose traces are shown in blue in B. (**C**) Derivation of the playback movie. We selected the fourth sniff-response during ethyl butyrate presentation as the basis for our movie (between vertical lines, magnified in D). Traces were deconvolved (deconv) with a 610 ms off-time constant to yield the red traces. fps: frames per second. (**D**) The temporal dynamics of the S+ movie between the anterior and posterior ROI differ (red traces), in that the anterior ROI turns on and off more slowly. The dynamics were removed for the S- movie by turning every pixel on and off at the same time to a fixed brightness level (green traces) that, integrated over time, equaled that of the dynamic pixels of the S+ movie (same AUC). Yellow traces show the mean (±s.d.) brightness averaged across the entire image. Frames were up-sampled from 25 fps (imaging rate) to 60 fps (movie frame rate). (**E**) Performance of mice on the playback movie task. Mice perform significantly above chance both before and after removal of the eyes (enucleation; *n* = 4 mice, 5–9 d each, *p*<0.00001).

In Paradigm 3 we aimed to establish directly the biological relevance of this ability to detect timing differences. We hence moved from synthetic biomimetic maps to a biological dynamic odor map recorded from the OB of a transgenic mouse in response to 0.7% ethyl butyrate (EB). We used a spatiotemporal map obtained from a GCAMP3-EMX mouse as the stimulus (virtual odor) to be projected (60 fps grey scale) onto the Thy-1 ChR2 mouse dorsal OB (see Fig. 4A–4D, S1 Text, and S1 Video). We chose a GCaMP3-EMX mouse as it provided signals of unprecedented quality (see approximately 10% dF/F in Fig. 4C).

Mice were indeed able to distinguish between the S+ OB-response movie, which contained the biological temporal information, and the static S- movie of same duration (Fig. 4E), devoid of the timing information (Fig. 4E, 74.8±2.0%; *p*<0.0001, above 50%, unpaired one-sided *t*-test, *n* = 4 mice, five to nine sessions per mouse). Clearly, if odor maps were read as static snapshots mice would not be able to distinguish between the two stimuli, because the spatial pattern of activation was identical.

Since the stimuli in Paradigm 3 were all sniff-triggered, there was a remote possibility that mice may be detecting the timing of the delayed glomeruli of the S+ stimuli relative to sniffing, as had been seen in the work by Rinberg and colleagues [13],[15]. Therefore, to eliminate the possibility that mice may be using the timing relative to the sniff phase as a potential cue, we introduced a uniformly distributed random jitter of 0–50 ms at the start of both S+ and S- trials (Fig. 3C, blue versus red lines). The four mice were indeed able to do the playback task (Fig. 4E). Additionally, no significant difference was revealed in the performance before and after the introduction of jitter (72.5±2.8% versus 73.8±1.8%, mean ± SEM, *p* = 0.61, unpaired *t*-test, *n* = 2 mice).

To control for any possible visual discrimination of the light stimuli, we enucleated (removed the eyes from) the mice. After recovery, mice were still able to perform with an accuracy of 76.5±5.2%, which was not significantly different from their performance before enucleation (*p* = 0.28, unpaired *t*-test, *n* = 4 mice, average of eight sessions per mouse) (Fig. 4E), showing that stimulus discrimination was independent of any visual signal and solely due to MTC activation. We ensured that we light-activated MTC in a physiologically relevant way (i.e. evoked activity that did not saturate) by having recorded MTC single unit activity in response to light of identical spectrum and brightness (Fig. 1). This also confirmed work by others [17] that spike onsets were tightly controlled by light. We therefore conclude that odors are represented by spatiotemporal dynamic maps and the timing information contained within this bulbar response can be used to disambiguate information pertaining to odor quality and, importantly, do so independently of the sniff cycle.

## Discussion

The bulbar odor response is rich in temporal information, associated not only with the presynaptic response pertaining to ORN input [8],[11] but also seen with the post-synaptic output neuron (MTC) responses [10],[22]. Mechanisms for temporal processing within the olfactory bulb include inhibitory gating by granule cells of mitral cells [23], as well as inhibition of input [24],[25] and output [26] at the glomerular level.

While studies by Mouly and colleagues electrically stimulate spatial glomerular activity patterns in rats [27]–[29], temporal odor coding has not been investigated beyond the question of whether discrimination depends on the phase of the sniff-cycle in which the stimulation occurs [27]. More recently it has been shown that the timing of presynaptic odor activation relative to the sniff cycle can be detected by mice down to 10 ms [13],[15]. Here we show timing of similar accuracy of odor discrimination is possible even without reference to the sniff cycle.

We suggest that the earliest activated glomeruli and MTC serve as a time reference "internal" to the OB. Such internal sniff signal-independent reference has previously also been suggested in the form of the whole-population MTC activity [30]. This is however not to dismiss a significant role for sniffing as a temporal reference in the olfactory bulb, and we propose that both sniffing as well as internal relative glomerular and MTC dynamics contribute information about time to the animal.

Because animals in Paradigm 3 were able to discriminate dynamic versus static patterns of equal duration, this suggests that the mechanism involved in the discrimination of Paradigm 2 is feasible based on the relative onset times of the ellipsoidal spot only and not the overall stimulus duration.

A recent study by Haddad et al. [14] provides neurophysiological evidence for the transmission of bulbar timing information to the piriform cortex. It is therefore plausible that temporal latencies, notably the order of glomerular activation [31], as encoded in the periphery are also part of the coding scheme in the piriform cortex. Such temporal processing may occur in our discrimination task, as timing in reference to sniff cycle phase at the level of principal neurons of the OB is not necessary and such temporal coding may be processed elsewhere.

Studies have shown that the precise locking of MC firing to the sniff cycle can facilitate ensemble olfactory coding [13],[32] perhaps by enabling synchronization across neurons [33]. However, it is important to note that this was not used as a cue in our paradigm, as shown by the lack of the effect of the stimulus onset jitter relative to the sniff onset. This implies that timing relative to sniff phase detection and timing relative to activation of other MTCs might be distinct signals used by mice in odor encoding.

Our stimulation paradigm directly targets principal MTCs to examine how temporal coding in this layer of the OB is required for odor discrimination. Given the average brightness of 2–5 mW/mm^2^ of the stimuli that were projected onto the dorsal OB, at 1 mm depth into the OB this optical power should already be at the neural activation threshold (0.5 mW/mm^2^), given that 480 nm light power is reduced by 90% by brain optical scatter at 1 mm distance from a light source [17]. Anatomically, the only nearby non-OB brain tissue is the prefrontal cortex. This area is more than this threshold distance of 1 mm away from our excited region [34]. So, light passing beyond the OB is unlikely to stimulate neurons if they were to be thy1-ChR2 positive, including known centrifugal modulatory inputs. By our own findings (Fig. 1) and others [17],[18] thy-1 driven expression is predominantly in output cells. To the extent that there is ChR2 expression that is Mitral/Tufted cell-independent, if any at all [17],[18], they are both lower in number and expression levels, as well as deeper (granule cell layer [GC]) than MTC, all strongly suggesting only a relatively minor population effect, if any at all. Furthermore, we confirm that potential visual inputs are not interfering with this discrimination task by using both a head-mounted "light funnel" to prevent light from reaching their eyes externally, as well as bilateral enucleation. Considering the unlikely stimulation of non-MTCs and confirmation of no visual stimuli interference, we conclude that the behavioral discriminations of high temporal resolution reported here depended entirely on the OB, and likely solely on MTCs.

Paradigm 3 has several limitations. First, we do not know whether the replay of imaged bulbar dynamics in Paradigm 3 generated a percept identical to that of the actual EB odorant that was used to generate the optical stimulus. To demonstrate this would require a discrimination task of the actual odor versus light-based spatiotemporal pattern replay. We feel this identity is not a requirement, however, as we intended to demonstrate that optical stimulation based on actual odor-evoked neural activity, in contrast to the biologically inspired but not biologically recorded stimuli, could also be successfully discriminated. Second, Paradigm 3 used only a static control stimulus to establish that MTC dynamics are detectable. We hypothesize that mice can also discriminate among stimuli with different sequences of MTC activation, but this remains untested. Third, the time-integrated brightness of the S+ and S- stimulus are identical (S1 Figure), and for them to generate equal total spike counts requires linearity of the brightness-firing rate relationship. There is some data, c.f. Fig. 2A in [17] and Supplementary Fig. 1A in [35], showing a fairly linear relationship between light intensity and photocurrents in MTC. For faithful playback of the original MTC activity, we also assumed a roughly linear relationship between firing rate and GCaMP3 activity, which was demonstrated in hippocampal cells in (c.f. Fig. 3B in [36]). These relationships should be corroborated by future studies across a population of MTCs.

One of the surprising results of our work is just how temporally precise the olfactory system can be with regard to recognizing the temporal dynamics among glomerular post-synaptic neurons. The auditory system is considered the most sensitive, followed by the touch and visual system in humans, with weber fractions (detection time/stimulus duration time) at 10%, 16%, and 20% respectively [37]. Our data show that the mouse olfactory system, with a weber constant of 13% (13 ms for the 100 ms model movies of Paradigm 2), is comparable to the vibrotactile sense.

For another major component of the flavor system, the gustatory system, it is found that the temporal characteristics of taste responses convey information about the quality and intensity of a taste stimulus [38],[39] at timescales greater than 250 ms [40]. Rats can respond in a taste-specific manner depending solely on the temporal stimulus pattern in the nucleus of the solitary tract [41]. The flavor system hence employs temporal information, albeit with the olfactory system acting at an approximately 20 times finer timescale.

The fine temporal structure of the odor-evoked response at the level of the OB principal MTCs is functionally significant for odor perception. Our behavioral confirmation of a temporal dimension to the decoding of OB odor maps, combined with timing information related to the sniff phase and the importance of the graded nature of individual presynaptic glomerular responses [13],[15], implies a maximized transient information flow rate through the olfactory system.

## Methods

### Overview

All the animals were treated according to the guidelines established by the U.S. National Institutes of Health (2011), and the experimental protocols were approved by the Institutional Animal Care and Use Committee of the John B. Pierce Laboratory. The John B. Pierce Laboratory is AAALAC accredited. Behavioral performance data and stimulus data are available in a permanent repository (http://dx.doi.org/10.5061/dryad.01br7) [42]. Thy1-ChR2 heterozygous mice and wild-type were implanted with headbars for head fixation and the skull dorsal to the OBs was thinned and coated with super glue for optical transparency. After at least 5 d of recovery and 2 d of water regulation (15 min/day) mice were adapted to head fixation in the pletysmography box. Mice were then trained to perform a go/no-go task to (1) detect an odorant; (2) discriminate two odorants; (3) detect the optical dynamic S+ stimulus; and (4) discriminate the static versus dynamic optical stimuli of Paradigm 1 (250 ms duration per spot, single presentation per trial, delay time brought down to mice's delay threshold); followed by (5) discriminate the static versus dynamic sniff-triggered optical stimuli of Paradigm 2 (100 ms spot duration, down to mice's delay threshold); and finally (6) to discriminate the replayed biological dynamic versus static OB response projection pattern in Paradigm 3. These OB patterns were based on the optical imaging of awake heterozygous GCamP3-EMX mice. Mitral/tufted cell electrophysiology was performed in anesthetized Thy1-CHr2 mice.

### Mice

Adult mice (60–400 d) were used in this study. Six C57BL/6 mice (Charles River, Wilmington) were used as controls. Our experimental group consisted of 10 Thy-1 ChR2 mice expressing channelrhodopsin in M/T cells of the OB [17]. Mice were water restricted for at least 2 d prior to the start of the training session. During water restriction, access to water was limited to 15 min every day. Food was available ad libitum. The weight of the mice was monitored daily. GCAMP3-EMX mice obtained by crossing a GCAMP3 reporter line (Jackson Laboratory, Maine) with EMX-CRE mice (donated by Drs. Robert Sachdev and David McCormick, Yale University, New Haven) were used for optical imaging experiments.

### Optical Window and Head Bolt

The mice were anesthetized with ketamine and dexdomitor (75–100 mg/kg and 0.5 mg/kg respectively, i.p.). Antisedan (0.5 mg/kg SC) was used for the reversal of the sedative effect. Toe-pinch reflex was checked before the start of the surgery as well as periodically during the surgery to ensure that the mouse was deeply anesthetized. The bone overlying the dorsal surface of the bulb was exposed, thinned and coated with cyanoacrylate glue to make the bone transparent. This yielded a ∼10 mm^2^ optical window which was clear for several months and was re-thinned when clarity was reduced. A head bolt for head fixation during the behavioral task was attached to the exposed skull using dental acrylic. Mice were allowed to recover for approximately 5 days before being put on water regulation in preparation for the start of training.

### Behavioral Tasks

The mice were trained on a go/no-go task where the trial types (S+ or S-) were chosen randomly. Mice were trained for at least ten blocks; each block consisted of 20 trials. Each trial lasted for 5 s, with a tone indicating the start of a trial. The stimulus (odor or light pattern/movie) was presented after a 1.8 s delay. The reward for a correct S+ lick (11 µl water) was available 200 ms after the start of stimulus presentation. Incorrect licks of S- were punished with 1 M NaCl (11 µl), time-out of 3 s, as well as an incorrect lick tone. Six seconds separated each trial. A vacuum tube along the lick spout sucked up any residual fluid before the start of a new trial.

Initially, head-fixed mice were trained to lick the lick spout when presented with S+ odor alone. Once the mice were accustomed to licking the lick spout for reward, the S- odor was introduced randomly. Training started with no additional ITI or salt punishment, so as not to discourage the mouse. Both were introduced 1–2 d later. Mice usually took 1–3 d to acquire the odor discrimination task and perform at >80% accuracy. Once they reached this stage, the stimulus was changed to the light patterns and movies.

Mice were first trained to perform a detection task (S+ versus no light). Once the mice were able to perform this task with >85% accuracy (usually within 3–4 d), S- was introduced. After task acquisition, the brightness was titrated down (by 50%–80%, from 100 mW, being 11 mW/mm^2^) to the minimum level still allowing approximately 90% correct responses (approximately 2–5 mW/mm^2^). We used a calibrated Thorlabs PM100D with the S121C sensor, set to 480 nm, for all optical power measurements.

For discrimination of the light patterns, three sets of behavioral paradigms were followed. Initially mice in Paradigm 1 were trained on a biomimetic model stimulus where a single movie (S+ or S-) was presented during the trial. The rostral set of four ellipsoid spots was presented for 250 ms and after a specific delay a caudal set of four ellipsoids was also presented for 250 ms. Once the mice were able to discriminate at an accuracy of 80% for a single block or 75% for 2 blocks (*p*<0.005), the delay was manually decreased by a single frame (by 16.7 ms, 1/60th of a second). Each ellipsoid spot spanned 593 µm (a–p) ×445 µm (m-l) (152×114 pixels). All eight spots combined made up 14.1% of the total 4×3 mm projected frame area. The anterior set of four spots was shifted 1993 µm anterior to the posterior set (Fig. 2A). The more medial (and anterior) spots were 542 µm apart from each other (m–l, edge–edge) and the more lateral (posterior) spots were 1,541 µm apart. Within each set of four spots, the anterior spots were shifted 667 µm anterior to the posterior spots.

Mice in Paradigm 2 were trained on sniff-triggered movies where the same model stimulus was presented approximately 10 ms after each sniff detected during a trial. In this paradigm, the rostral and caudal sets of ellipsoids were each presented for a duration of 100 ms. An automated procedure decreased the delay from 250 ms down in 17 ms (1/60 s, one video frame) increments whenever the subject was able to respond correctly in eight consecutive trials (*p* = 0.004 by chance), or increased delay during eight consecutive errors. After mice progressed to single frame delay, subframe delay (i.e., at 1,440 fps instead of 60 fps) adjustments of 25% were made. Once mice got to a stage where they reached subframe delays (below 17 ms) reliably, all training days henceforth were included in our analysis. Occasionally, even after reaching the above criteria, mice failed to perform the task. Such days, identified by when they did not progress beyond discriminating a single frame (17 ms), were excluded from analysis.

In both groups, temporal discrimination threshold was defined as the minimum delay allowing 75% correct responses during a daily session.

In Paradigm 3, mice were trained to discriminate between a pre-recorded OB odor response movie as the S+ and a spatially identical S- movie, devoid of timing information (S1 Video). The movies were scaled to match the size of the dorsal OB window and presented at 7–10 mW overall brightness. A uniformly distributed random jitter of 0–50 ms was introduced at the start of both S+ and S- trials to eliminate any potential sniff timing cue. Once mice were reliably able to discriminate the playback odor movie with at least 70% accuracy for a minimum of 3 d, it was assumed that they had acquired the task. All sessions henceforth were used for data analysis. Any day when mice failed to lick for more than 10% throughout the training session (at least five blocks) was excluded from analysis, as it was assumed that mice were not motivated enough.

To ensure that discrimination was only via activation of channelrhodopsin in the MTCs and not due to visual detection of the light stimulus, we introduced the following measures. First, to mask any light that may reach the retina (only approximately 1 mm apart from the OB by the orbital bone) we presented intense blue 480 nm LED, located 4 mm lateral to each eye, starting 100 ms before OB stimulus onset, for the entire trial duration. This LED mask consisted of a constant brightness level (approximately 10 mW through mouse-pupil–sized pinhole at eye distance) summed with temporal white noise (0–10 mW). Second, a black ABS "light funnel" was implanted on their OB window to prevent light from reaching their eyes externally (Fig. 1). Third, at the end of Paradigm 3 mice were blinded by bilateral enucleation, thereby eliminating any visual cue. Mice were then once again evaluated on the light discrimination task. All data are reported ±SEM.

### Experimental Setup

The stimulation setup was organized around a modified Olympus BX50WI microscope. The light source consisted of five 700 mW 455 nm lasers aimed at a 6 mm OD liquid light guide. The guide entered into the port of a custom-made DLP projector (Zinterscope, Guilford, CT), which contained the Texas Instruments D4100 0.7 XGA 1024×768 micro-mirror device (Fig. 1). A DVI to DMD (D2D) Interface board (Digital Light Innovations), supporting 24- bit binary expansion (60×24 = 1,440 fps, Paradigms 1 and 2) and 8-bit grey scale (60 fps, Paradigm 3), was plugged into the DLP board. Images were projected on to an Olympus UPlanFLN 4× n.a. 0.13 objective, yielding an image size of 3×4 mm that was focused onto the dorsal OB. Lasers were TTL-triggered via PWM with a 40 kHz cycle. The maximum brightness projected onto the OB, when lasers were driven at a 90% duty cycle and the entire DMD image turned on, was 100 mW. Frame timing was validated using a phototransistor and oscilloscope. For consistency across daily sessions, we centered the dorsal OB on a plus-shaped light pattern using an xy-stage onto which the mouse box was mounted.

Sniffing was measured with a whole-body plethysmograph [20], where the mouse was enclosed in an acrylic box (187 cm^3^). Bias airflow was provided by constant flow rate of 0.7 l/min house air and a Buxco vacuum (Wilmington, NC). The lid had a coverslip-covered cut-out above the OB and a black foam ring that sealed in the light entering the black OB funnel. The box pressure was transduced by a sensor (Buxco TRD5700), filtered 0.1–100 Hz, and amplified 100,000× (100× via a generic amplifier, 1,000× via a WPI DAM50). Licking was measured by a contact lickometer (MedAssociates ENV250). Two controller boards (NI USB-6259) interfaced the hardware to the PC Labview environment. For the sniff-triggered movie paradigm, Python was used to detect sniffs on one PC and trigger another ("movie server") PC over TCPIP to generate the movies with constant approximately 10 ms delay and an optional uniformly distributed random jitter of 0–50 ms (Fig. 3C). The movies, onset timing, and delay adjustment was controlled in Python, and to ensure that each movie was detected as a separate entity, triggers within 125 ms of the previous one were ignored. Labview was used for the overall go/no-go task control (Fig. 1).

### In Vivo Electrophysiology

Thy-1ChR2 mice were anesthetized with ketamine (100 mg/kg i.p.) and xylazine (10 mg/kg i.p.). The anesthesia was maintained with boosters as needed. Atropine (0.04 mg/kg i.p.) was administered every 2 h to improve breathing by reducing secretions in the respiratory tract. The animals' body temperature was maintained with a heating pad set at 37°C. Lidocaine was applied prior to incisions. Craniotomy was preformed over both olfactory bulbs. The bulbs were covered with a 2% agar and saline solution to minimize pulsations. In vivo extracellular recordings were made in the mitral cell layer. Extracellular electrodes in glass micropipettes (3–6 MΩ) containing 2.5 M potassium citrate were used. Recordings were collected with RZ5 Bioamp Processor and RA16PA 16 Channel Medusa Preamp amplifier (Tucker-Davis Technologies). Cells were stimulated optically using a Digital Micromirror Device (Texas Instruments) built into a custom projector (Zinterscope, CT). The light stimulus was a 300×300 pixel square that was centered on the recording site. The intensity of the light stimulus was controlled manually by adjusting the laser brightness to match intensities used in behavioral experiments.

### Immunocytochemistry

Mice were killed and decapitated. The olfactory bulb was removed and kept in 16% paraformaldehyde overnight. 100 µm coronal sections were made on a vibratome (Leica) and were washed in 0.1% PBS. Some sections were incubated with primary antibodies mouse anti-GFP (Molecular Probes, OR) and rabbit anti-5HT2A (Abcam, MA). Secondary antibodies used were alexa fluor 555 goat antimouse and alexa fluor 488 goat antirabbit (Molecular Probes, OR). Sections were mounted on a slide with mounting medium containing DAPI (Vector Labs, CA). Sections were viewed on a three-channel laser scanning Zeiss confocal microscope 710.

### Optical Imaging and Playback Movie for Paradigm 3

Optical calcium signals from the dorsal OB were recorded using a CCD camera (Redshirt Imaging) with 256×256 pixel resolution and a frame rate of 25 Hz. The epifluorescence microscope used was a custom-made tandem-lens type with 1× magnification (F50/0.95). A high-power LED (Luxeon LXHL-PE09, Philips Lumileds) driven by a linear DC power supply acted as the light source. A custom-made DC amplifier (based on a linear Apex power operational amplifier; Cirrus Logic) powered a peltier (OT2.0-31-F1; Melcor) device, onto which the LED was glued. The LED-cooling peltier current was proportional to the LED current, yielding a stable illumination. The fluorescence filter set used was FF01-475/50-50 (excitation filter), LP515 (dichroic), and LP530 (emission filter; Semrock). This system provided fast imaging capabilities, a large field of view, and low noise.

Data were imported into Matlab R2013A. Raw images were converted to images representing the relative change in fluorescence (%Δ*F*/*F*) in each pixel and frame. Each trace was bandpass filtered (0.1–7.5 Hz, 4th order Butterworth) using. To get an estimate of the firing rate from the slow calcium signal, the ΔF/F signals were deconvolved using a time constant of 610 ms [43], using the following Matlab code: "kernel = exp((0:-1:-kernellength)/(tau * Sampling_Rate)" and "*deconv_tr  =  deconv([filt_tr kernelpad], kernel)*". Eleven frames (105–115) were selected as sniff-response for further preparation for playback (Fig. 4). The area outside the OB window was masked (set to 0). The 11 frames that were acquired at 25 fps were resampled to our projection rate of 60 fps yielding 27 frames, after subtracting out any offset present in the first frame. Values <0.01% dF/F were then set to 0. This S+ movie was next de-pixelated by convolving with a large spatial kernel. To determine the onset and offset times of the static S- movie we determined the frame at which the response rose to above 10% of peak and subsequent decline to below 10%, for all traces with a minimum response peak of 1.5% dF/F (*n* = 15,819 traces, 23% of 256×256 pixels). The onset frame was set as frame 2 (mean  = 2.2, median  = 2) and offset frame as frame 23 (mean  = 22.2, median  = 23). Each on-frame of S- movie was subsequently created as the frame based on the Area Under the Curve (AUC) of each S+ trace divided by 22 (number of on-frames). Thus, each pixel in the static S- spatial pattern had the same AUC as in the S+ movie (S1 Figure). Therefore the overall intensity over time was identical for both S+ and S-. Last, the movies were rotated and normalized between 0–255 by the min (0%) and max dF/F (5.5%) of the S+ movie (S1 Video).

## Supporting Information

S1 Figure **Total brightness of the movies used in the playback experiment (Paradigm 3).** The AUC of each pixel is identical for the S+ (left) and S- (right) movie to avoid cues unrelated to timing. The brightness of the static S- movie was scaled to obtain this result.
(TIF)

S1 Text **Supplementary information.**
(DOC)

S1 Video **The two movies used in the playback experiment (Paradigm 3).** A movie was presented for each sniff during a 3.2 s period on each rewarded or unrewarded trial. On the left is the unaltered dynamic S+ rewarded "go" movie, based directly on OB activity optically imaged from an awake GCaMP3-EMX mouse. During the 3.2 s trial period the S+ movie-evoked bulbar activity in ChR2 mice recapitulates the MC activity that would have occurred in the dorsal OB for each sniff of ethyl butyrate in the GCaMP3-EMX mouse. It shows complex dynamics, which are removed from the S- "no-go" movie on the right. The static S- movie on the right consists of a repetition of a single image based on the time-integrated fluorescence changes of the S+ movie (see S1 Figure). The scale is in percent dF/F. Slowed 4× for clarity.
(MOV)

## Abbreviations

AUC: Area Under the Curve
DLP: Digital Light Processor
DMD: Digital Micromirror Device
DVI: Digital Video Interface
EB: ethyl butyrate
fps: frames per second
GC: granule cell layer
MTC: mitral/tufted cell
OB: olfactory bulb
OE: olfactory epithelium
PSTH: Post Stimulus Time Histogram
Regions Of Interest: ROIs
YFP: Yellow Fluorescent Protein
