# Supplementary figures and images for: Perception of Odors Linked to Precise Timing in the Olfactory System
Source: PLoS Biol. 2014 Dec 16;12(12):e1002021. doi: 10.1371/journal.pbio.1002021 (PMC4267717; doi:10.1371/journal.pbio.1002021)

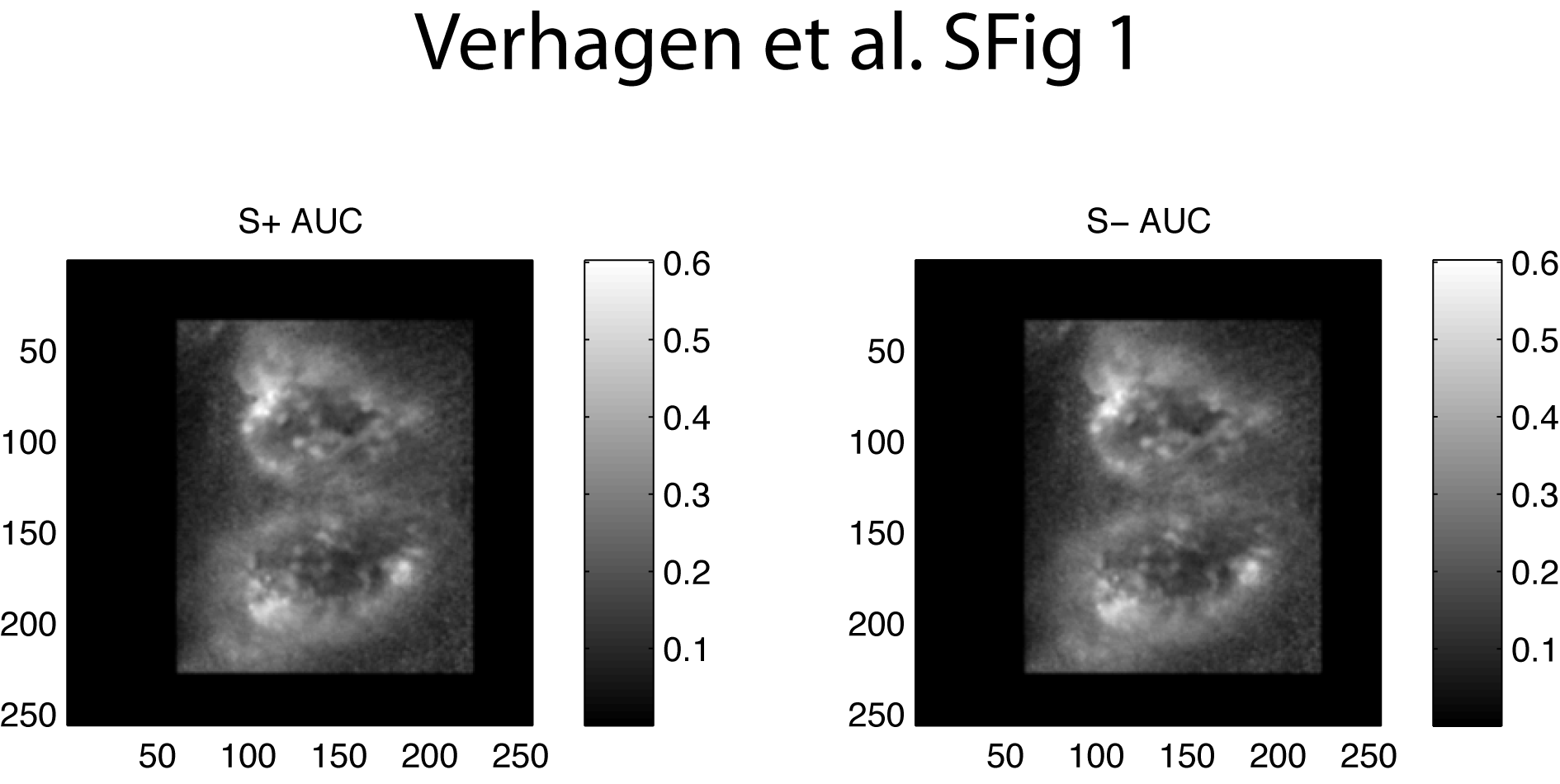

Supplement: S1 Figure — Total brightness of the movies used in the playback experiment (Paradigm 3). The AUC of each pixel is identical for the S+ (left) and S- (right) movie to avoid cues unrelated to timing. The brightness of the static S- movie was scaled to obtain this result. (TIF) [file pbio.1002021.s001.tif]
