# Supplementary material for: Perception of Odors Linked to Precise Timing in the Olfactory System
Source: PLoS Biol. 2014 Dec 16;12(12):e1002021. doi: 10.1371/journal.pbio.1002021 (PMC4267717; doi:10.1371/journal.pbio.1002021)
Supplement: S1 Text — Supplementary information. (DOC) [file pbio.1002021.s002.doc]

Perception of odors linked to precise timing in the olfactory system

**Authors:** Michelle R. Rebello, Thomas S. McTavish, David C. Willhite, Shaina M. Short, Gordon M. Shepherd, Justus V. Verhagen.

**Text S1**

We have shown that the activation of glomeruli relative to each other is an important behavioural cue in mice. In our study we control the latencies of mitral cell firing relative to one another and find that mice can detect subtle differences down to 13 milliseconds. Control mice not expressing ChR2 are unable to discriminate the light patterns. We establish the physiological relevance of this temporal detection by playing back temporally dynamic odour maps recorded from mice.

The OB light patterns in paradigm 1 and 2 were chosen bearing in mind the following criteria- first; odorants generally activate several glomeruli in a bilaterally symmetric pattern . There is also a second axis of symmetry within each bulb running posterior-lateral to anterior-medial and finally, glomeruli of related chemical specificity cluster together . We therefore created a representative response map, but of no particular odor, which could be used as a model stimulus reflecting a generalized odour response in the dorsal OB.

In Paradigm 2, the sniffing frequency in mice ranged from 3-7 Hz and on average 9.4±0.8 movies were presented per 5 second trial with an inter-stimulus interval of 339±37.2ms. The average response time (on S+ trials only) of mice was 899.7 ±123.1ms (mean ±SD) and mice “sniffed” 3.9 ±0.5 movies before licking for reward. An automated procedure decreased the delay from 250ms down in 17ms (a single video frame) increments whenever the subject was able to respond correctly in 8 consecutive trials (p=0.004 by chance), or increased delay during 8 consecutive errors (Incorrect licks or rejects). Once mice progressed to single frame delay, sub-frame delay adjustments of 25% were made. Therefore, unlike the previous paradigm, where each trial had a single presentation of an S+ or a S-, a stimulus was now presented with every sniff during the trial to mimic inhalation of an odour during sniffing (**Figure 3C**). There was no correlation between the delay and reaction time for S+ trials on each day (r^2^<0.01), irrespective of whether reaction time was determined from the start or end of the S+ movie. In our tasks the mice have no advantage in responding earlier, as the timing of the trial's start and end is fixed. They may become more certain as they wait and sniff more stimuli, at no cost.

GCAMP3-EMX mice were used for calcium imaging of the dorsal OB. GCAMP3-EMX mice have been shown to express GCAMP3 in multiple GABAergic interneurons including sub-populations of periglomerular cells and the superficial granular cell layer in the OB (**Figure 4A**) . These mice showed robust signals in response to odours (**Figure 4B,C**). It has been previously shown that spatial patterns of odorant-evoked calcium signals from various cell types in the OB (OSNs, mitral cells, periglomerular and short axon cells) are similar and show regional organization of response latencies . The spatial distribution as well as temporal latency associated with glomeruli also tends to be conserved between mice .

EB was chosen as it is known to excite most glomeruli in the dorsal OB , this ensured that the dorsal odour movie represented a more or less complete odour map. An odour movie recording was selected which showed distinct temporal dynamics between anterior and posterior glomeruli (**Figure 4B-D, also see Video S1**). To get an estimate of the firing rate from the slow calcium signal, the ΔF/F signals were deconvolved using a time constant of 610ms . The S+ movie consisting of 11 frames representing a single sniff was thresholded, de-pixelated and upsampled to 60 fps and 27 frames (8 bit grey-scale) (**Figure 4D**). The dynamics were removed for the S- movie by turning every pixel on and off at the same time to a fixed brightness level. Each pixel in the static S- spatial pattern had the same area under curve (AUC) as the corresponding pixel of the S+ movie (**Figure S1**). Therefore, just as our previous two behavioural paradigms, the time-integrated power was identical for both S+ and S- for each pixel. The on/off timing of the static S- was the average of the onset/offset frame of the S+ movie (T_10_,, on from frame 2-23) (**see Methods, Figure 4 and Video S1)**.

References:

1. Vassar R, Chao SK, Sitcheran R, Nunez JM, Vosshall LB, et al. (1994) Topographic organization of sensory projections to the olfactory bulb. Cell 79: 981-991.

2. Johnson BA, Woo CC, Hingco EE, Pham KL, Leon M (1999) Multidimensional chemotopic responses to n-aliphatic acid odorants in the rat olfactory bulb. The Journal of comparative neurology 409: 529-548.

3. Belluscio L, Katz LC (2001) Symmetry, stereotypy, and topography of odorant representations in mouse olfactory bulbs. The Journal of neuroscience : the official journal of the Society for Neuroscience 21: 2113-2122.

4. Mori K, Takahashi YK, Igarashi KM, Yamaguchi M (2006) Maps of odorant molecular features in the Mammalian olfactory bulb. Physiological reviews 86: 409-433.

5. Kohwi M, Petryniak MA, Long JE, Ekker M, Obata K, et al. (2007) A subpopulation of olfactory bulb GABAergic interneurons is derived from Emx1- and Dlx5/6-expressing progenitors. The Journal of neuroscience : the official journal of the Society for Neuroscience 27: 6878-6891.

6. Wachowiak M, Economo MN, Diaz-Quesada M, Brunert D, Wesson DW, et al. (2013) Optical dissection of odor information processing in vivo using GCaMPs expressed in specified cell types of the olfactory bulb. The Journal of neuroscience : the official journal of the Society for Neuroscience 33: 5285-5300.

7. Soucy ER, Albeanu DF, Fantana AL, Murthy VN, Meister M (2009) Precision and diversity in an odor map on the olfactory bulb. Nature neuroscience 12: 210-220.

8. Spors H, Wachowiak M, Cohen LB, Friedrich RW (2006) Temporal dynamics and latency patterns of receptor neuron input to the olfactory bulb. The Journal of neuroscience : the official journal of the Society for Neuroscience 26: 1247-1259.

9. Johnson BA, Leon M (2007) Chemotopic odorant coding in a mammalian olfactory system. The Journal of comparative neurology 503: 1-34.

10. Tian L, Hires SA, Mao T, Huber D, Chiappe ME, et al. (2009) Imaging neural activity in worms, flies and mice with improved GCaMP calcium indicators. Nature methods 6: 875-881.
